# Engaging research with policy and action: what are the challenges of responding to zoonotic disease in Africa?

**DOI:** 10.1098/rstb.2016.0172

**Published:** 2017-06-05

**Authors:** Kevin Louis Bardosh, Jake Cornwall Scoones, Delia Grace, Gladys Kalema-Zikusoka, Kate E. Jones, Katinka de Balogh, David Waltner-Toews, Bernard Bett, Susan C. Welburn, Elizabeth Mumford, Vupenyu Dzingirai

**Affiliations:** 1 Department of Anthropology and Emerging Pathogens Institute, University of Florida, 2055 Mowry Road, Gainesville, FL 32610, USA; 2 Clare College, Cambridge CB2 1TL, UK; 3 International Livestock Research Institute, PO Box 30709, Nairobi, Kenya; 4 Conservation Through Public Health, Plot 3 Mapeera Lane, Entebbe PO Box 75298 Clock Towers, Kampala, Uganda; 5 Centre for Biodiversity and Environment Research, Department of Genetics, Evolution and Environment, University College London, Gower Street, London WC1E 6BT, UK; 6 Institute of Zoology, Zoological Society of London, Regent's Park, London NW1 4RY, UK; 7 Regional Office for Asia and the Pacific, Food and Agriculture Organization of the United Nations (FAO), 39 Phra Atit Road, Phranakon, Bangkok 10200, Thailand; 8 Department of Population Medicine, Ontario Veterinary College, University of Guelph, Guelph, Ontario, Canada N1G 2W1; 9 Division of Pathway Medicine and Centre for Infectious Diseases, School of Biomedical Sciences, College of Medicine and Veterinary Medicine, University of Edinburgh, Edinburgh, UK; 10 Department of Country Health Emergency Preparedness and IHR, World Health Organization, 1211 Geneva 27, Switzerland; 11 Centre for Applied Social Science, University of Zimbabwe, MP167 Mt Pleasant, Harare, Zimbabwe

**Keywords:** One Health, zoonotic disease, policy, practice

## Abstract

Zoonotic diseases will maintain a high level of public policy attention in the coming decades. From the spectre of a global pandemic to anxieties over agricultural change, urbanization, social inequality and threats to natural ecosystems, effectively preparing and responding to endemic and emerging diseases will require technological, institutional and social innovation. Much current discussion emphasizes the need for a ‘One Health’ approach: bridging disciplines and sectors to tackle these complex dynamics. However, as attention has increased, so too has an appreciation of the practical challenges in linking multi-disciplinary, multi-sectoral research with policy, action and impact. In this commentary paper, we reflect on these issues with particular reference to the African sub-continent. We structure the themes of our analysis on the existing literature, expert opinion and 11 interviews with leading One Health scholars and practitioners, conducted at an international symposium in 2016. We highlight a variety of challenges in research and knowledge production, in the difficult terrain of implementation and outreach, and in the politicized nature of decision-making and priority setting. We then turn our attention to a number of strategies that might help reconfigure current pathways and accepted norms of practice. These include: (i) challenging scientific expertise; (ii) strengthening national multi-sectoral coordination; (iii) building on what works; and (iv) re-framing policy narratives. We argue that bridging the research-policy-action interface in Africa, and better connecting zoonoses, ecosystems and well-being in the twenty-first century, will ultimately require greater attention to the democratization of science and public policy.

This article is part of the themed issue ‘One Health for a changing world: zoonoses, ecosystems and human well-being’.

## 1. Introduction

We are coming to the time when we have to say: ‘Where is [a] One Health [approach to zoonotic disease] useful?’…It has been very successful in many respects. But it hasn't and isn't making a big difference in the ‘real-world’.

—Delia Grace, International Livestock Research Institute, Kenya

In our contemporary hyper-globalized world, anxieties surrounding zoonotic diseases have risen sharply in the public policy arena, with Africa featured prominently in these debates [1]. Pathogens spread between animals and people draw the interests of high-tech science and frequent the corridors of global power, tied to concerns over international trade and social order under the rubric of ‘global health security’ [2]. They are also an integral part of the wider global health and development agenda, central to food security, health and livelihoods for pastoralists, peasant farmers, ethnic minorities and other marginalized groups [3,4].

Where multiple, overlapping global crises compete for attention, quantification of the significant health and economic burden of these pathogenic threats forms the mainstay of public health discourse. The endemic zoonotic diseases (such as leptospirosis, cysticercosis, zoonotic tuberculosis, rabies, leishmaniasis, brucellosis and others) are estimated to cause more than 2.2 million human deaths and 2.4 billion cases of illness annually [5], disproportionately affecting the poor in the Global South. With additional health impacts, 75% of all emerging diseases are zoonotic in origin, including Ebola, West Nile and Nipah virus infections, SARS and zoonotic influenza. These pathogens negatively influence international trade and tourism [6]; a severe global pandemic, most likely emerging from an animal ‘spillover’ event, has the potential to kill 180 to 360 million people and reduce global GDP by as much as 10% in the first year alone, as estimated in Nahal & Ma [7].

Africa remains at the core of these debates in the twenty-first century [8]. The human population is estimated to soon double on the world's second largest continent, reaching some 2.8 billion by 2060 (or 25% of the world's population) [9]. With unprecedented rates of population movement and concentration in urban centres and megacities, new rapidly transmissible disease dynamics will emerge. This will be influenced by the increased demand for pigs, chickens, milk and other animal products, and changes in both extensive and intensive livestock production systems [10]. Climate change projections also show disproportionate effects in Africa, with upwards of a 20% reduction in crop yields by 2050 due to heat stress, drought and flooding events [11]. Environmental change, often considered a major driver of disease emergence, is ubiquitous with negative trends frequently discussed in terms of significant deforestation, soil erosion, desertification, wetland degradation and species extinctions [12].

These demographic, economic, environmental and climatic changes will influence the disease ecology of zoonotic pathogens. The implications for health and ecosystems are predicted to be considerable and effective mitigation of these impacts, moving from a passive to proactive approach, will demand something new—a fundamentally different approach. While other conceptual approaches exist for addressing the many forces leading to zoonotic disease emergence and transmission, one of the most widely promoted internationally is a ‘One Health’ approach: working across disciplines and sectors to tackle complex, interconnected human–animal–ecosystem disease problems. One Health has been, to a varying degree, embraced by donors, scholars, civil society groups, governments and the three international organizations (known as the Tripartite) mandated to address health and disease in animals and humans: the World Organisation for Animal Health (OIE), The World Health Organization (WHO) and the Food and Agriculture Organization of the United Nations (FAO). Scholars and practitioners argue for greater integration between the disciplines of ecology, conservation, public health, agriculture, veterinary science and social science, and for the initiation of major reforms in our current global and local systems of surveillance, preparedness prevention and control [1,13–15].

Much has been made of using a One Health approach to tackle both endemic and emerging zoonotic diseases [1,15]. As a unifying ‘boundary object’ [16], the fluidity of the One Health concept is at times vague, and risks dilution and appropriation [17].^1^ How can we ‘do’ One Health? What does this mean in practical terms for local people? How useful is the label? What should we avoid doing? Is integration and multi-sectoralism always so useful and beneficial? What negotiations are involved, and institutional dynamics?

In this paper, we reflect on these questions. Our aim is to go beyond the perhaps unrealistic rhetoric of unity and holism, and ask: what does One Health in the ‘real world’ mean? And what needs to be done to better link research with policy and action for impact across sectors? To advance our analysis, we posed these two questions to 11 leading scientific experts at the high-level symposium, ‘One Health in the Real World: Zoonoses, Ecosystems and Well-being’, held at the London Zoological Society in 2016 (see: https://www.zsl.org/science/whats-on/one-health-for-the-real-world-zoonoses-ecosystems-and-wellbeing). This symposium was organized as part of the Dynamic Drivers of Disease in Africa consortium (DDDAC), a major UK-funded One Health consortium of social and natural scientists from 21 institutions in Africa, Europe and America (see: http://steps-centre.org/project/drivers_of_disease/). The major themes discussed in this paper were first identified, through standard qualitative coding analysis, in the 11 video interviews provided alongside this paper (see https://figshare.com/projects/Engaging_Research_with_Policy_and_Action_What_are_the_Challenges_of_Responding_to_Zoonotic_Disease_in_Africa_/19222). Our analysis was then extended through reference to the wider literature and the professional experiences of the authors, who have extensive experience working in Africa, and elsewhere, on zoonotic disease research, programmes and policy. Hence, the paper reflects a compilation of a high-level consensus on new ways forward for a One Health approach to zoonotic disease in Africa, with a strong social science orientation.^2^

Although the challenges and questions about One Health implementation are by no means restricted to Africa, the continent has attracted a disproportionate amount of attention. Kamani *et al*. [18], for example, have argued that One Health is ‘a concept led by Africa, with global benefits.’ In this sense, nascent veterinary and medical institutional infrastructures, so the authors continue, may offer an opportunity to build new integrated platforms and networks from ‘the ground-up’—a sort of institutional ‘leapfrogging’—with significant benefit for local people dependent on natural resources [18].

In the following sections, we explore contemporary challenges in zoonotic disease research, policy and action, and then turn our attention to specific strategies that can help reconfigure current orthodoxies, and better realize One Health in practical terms in the African context and beyond.

## 2. Defining the problem: knowledge, politics and capacities

### (a) The politics of knowledge

A central concern for One Health has been disciplinary integration, including the important role of new knowledge generation [1]. This narrative includes a defining emphasis on the need to gather evidence to influence decision-makers. A major focus has been on building the ‘evidence-base’ for more holistic understandings of disease ecology and burden [19], through epidemiological and socio-economic research: where are the pathogens? How do they circulate? What impacts do they have on health and livelihoods? A second strand of research, albeit much less promoted, discusses the important role of understanding systems of preparedness and intervention, why sectoral and disciplinary silos exist and how administrative structures and bureaucracies work. This has a clear utilitarian value; the hope is to use such knowledge, of systems and capacities, to modify or leverage institutional cultures in control efforts [20,21].

Both of these efforts are located in a third, and more general, emerging trend. This borrows from various other theoretical and conceptual approaches, including ecosystems approaches to health (also known as EcoHealth), and aims to understand complexity and systems dynamics across temporal and spatial scales. The drive is towards holism and change [22]. David Waltner-Toews, past president of Veterinarians Without Borders, and an important scholar in the EcoHealth field, summed up this sentiment in the following statement:

The real-world … is a complex set of interactions … a whole set of ecological, social and economic interactions and feedback loops … One Health … [is about] begin[ning] to think about the health of this whole complex mess, when we've got non-linear dynamics, we've got feedback loops: it's not a big machine or a computer where you can change a few parts…and everything is fine. One Health is a way to…hone our peripheral vision … [understanding and engaging] that whole set of interactions. (See electronic supplementary material.)

Complex entanglements—feedback loops, non-linear dynamics, cascade effects at different scales—make it hard to discern how specific overlapping, human–animal–ecosystem changes—and their political and cultural contexts—will affect specific disease ecologies. Hence, a dominant emphasis has been on advancing new methodological tools to understand these processes, attempting to integrate disciplinary understandings more fruitfully—for example, modelling approaches that account for combined climate and environmental change, land-use patterns and, for recently, socio-cultural and behavioural processes [22,23]. The African Livestock Futures project is a good, practically focused example of this trend, which aims to provide policy recommendations based on projected future changes across systems and scales [10].

While intellectually fruitful, in many cases the implicit assumption is that better research leads to more actionable knowledge. This is, in many cases, a highly questionable conclusion that lies at the heart of the current translational research crisis in academia, think tanks and government: the fact that much scholarly research lacks clear utilitarian value and/or languishes due to weak institutional and organizational pathways to application [24,25]. There is a major disconnect between how the research community generates knowledge, and the types of information channels non-academic audiences need, or can put into practice. The mechanisms to translate knowledge into process are frequently weak and overlooked. With significant front-line experience working at the global level and in Africa, Katinka de Balogh from the Food and Agriculture Organization of the United Nations (FAO), summarized this problem as follows:

We see often that research is being done in a kind of isolation. At university, the students [are] looking for a project [or there is a funding opportunity]. You think this … might be of interest. You do your research. But then often these research results are just published in a publication … [and] does not get into the real world. [There is no] translat[ion of] these findings into viable action … [we need to find new] mechanism[s] … to translate findings … to develop [zoonotic] disease control plans. (See electronic supplementary material.)

Conventional views on the linkage between research knowledge and action, such as the ‘trickle down’ effect and ‘technology transfer’, often rely on hierarchical and technically orientated approaches in implementation that, despite decades of critique, have remained dominant in science and policy [24,25]. This includes much current One Health literature [1], embedded within the current norms of academia where reward systems frequently limit opportunities to link scientific research with practical dissemination, local implementation and hence societal impact. Research is frequently not demand driven. While cognitive frames and priorities are important, African scholars have further located these failures in a lack of basic funding, mentorship and capacity issues at the university level that maintains these disconnections [26–28].

Transcending disciplinary ‘silos’ in research, then, is not only about developing new metrics, new methodologies and greater mixing of disciplines, but also about the politics of knowledge. *For whom* is knowledge being generated and *how* is it being used? This is not to deny the significant way that advancing research knowledge, by itself, contributes to the critical mass of knowledge and can generate positive zoonosis surveillance, prevention and control efforts. Knowledge accumulation and conceptual trends take time to develop and are based on cumulative synthesis; this is important. However, there are limitations to the ‘knowledge-for-knowledge-sake’ agenda that need to also be accounted for.

Disease models loom large in these debates due to their frequent centrality in policymaking. As Christley *et al*. [29] note, models are complex assemblages built on different levels of uncertainty; their usability is a product of the networks and discourses that surround them and the functional value they provide. This makes many scientists somewhat uncomfortable in actively engaging the policy world—it is difficult to communicate the intricacies of models and their uncertainties, especially when there is an expedient need for concrete facts and information to make decisions, as during an epidemic. Kate Jones, an expert zoonotic disease modeller from University College London and the Zoological Society of London, highlighted this issue in an interview:

Models … are [often] interpreted as the truth. They have some kind of authority over … people … [but] the uncertainties and the assumptions around those models aren't often discussed, and [it is] actually very difficult to communicate the uncertainties [to policymakers] … [leading the models themselves to] get misunderstood and not applied [properly] … . (See electronic supplementary material.)

The mismatch in professional and cultural interests and values between researchers and policymakers can be a significant barrier to linking sound knowledge with effective action and salient policy. Researchers benefit the most and gain the most prestige from publishing papers presenting new knowledge and innovative concepts. Knowledge production prioritizes ‘global’ audiences in the most prestigious scientific journals [30], and downplays local information sharing and community-based activities. There are few direct incentive and reward systems to encourage engaging in the more difficult, long-term and politically fraught translation of such knowledge into action or policy on the ground for local people [1]. The reward systems for research, therefore, tend to marginalize the needs of the marginalized. Research is done, and the benefits accrue in the number of publications, citations and future research grants obtained.

### (b) Implementation challenges

Current research norms and recommendations for change, however, need to consider the systemic challenges in local implementation systems, structures and capacities in Africa. These are essential to knowledge flow and application. One Health has struggled to be operationalized in practical terms due, in large part, to the nature of existing resource-limited systems [1].

Understanding the challenge of creating local One Health systems should be grounded in socio-historical-political contexts. African health systems have suffered extreme neglect since the colonial era and through periods of structural adjustment [31]. State capacities are often limited, giving rise to an amorphous and ill-defined mixture of public and private systems [32]. Disease outbreaks are regularly addressed by central governments, but most services are decentralized. This generates conflicts between the centre and periphery in planning, funding and implementation [33]. In current veterinary and public health sectors, government providers pursue private practice alongside their public roles, casual drug sellers provide unregulated services, and traditional and modern medicine intermingle. Remote regions, including many biodiversity ‘hotspots’ where pandemic spillover events are predicted to most likely begin, remain ‘non-state regions’ that lack essential government services [1]. Basic sanitation and water infrastructure, and access to quality pastures, agricultural inputs and markets remain weak and fragile.

One Health is often assumed to be a government responsibility, but in the context of weak or absent states, the responsibility for One Health policy and implementation are unclear. Who is responsible? And how can pilot studies be scaled-up and integrated into human health and veterinary systems? Delia Grace, Program Manager for Food Safety Zoonoses at the International Livestock Research Institute (ILRI) based in Kenya, which is part of the Consultative Group for International Agricultural Research (CGIAR), summed up the problem as follows:

We have lots of case studies [on One Health] … where you have … One Health at community levels, where vets and medics have come together to deliver vaccines for children and animals [for example] … but as they say ‘pilots never fail and pilots never scale.’ These make very nice case studies in reports, and people find them interesting and compelling, but obviously not compelling enough to change how we do business … . (See electronic supplementary material.)

Part of this disjunction involves relationships, and different interests and power, between organizations and institutions. At the implementation level, we find multiple fragmentations between official policy documents and their translation into local interests, capacities and infrastructures. Millstone *et al*.'s [33] critical social science analysis of Rift Valley fever (RVF) policymaking in Kenya is informative. It shows how disconnects between policy aspirations for RVF vaccination stockpiles and surveillance knowledge and pastoralist realities and capacities were driven by divergences between lower and higher tier policy stakeholders. There is often a lack of communication, and cultural divides, between groups in the capital and remote districts. Another informative example is given by Smith *et al*. [34] working in Uganda; they argue that donor-driven development initiatives for human and animal trypanosomiasis tend to avoid the African state, despite the rhetoric of ‘partnership’ and ‘collaboration.’ This severely compromises the ability to build long-term capacity for implementation by marginalizing processes of institutional memory, legitimacy and coordination.

Bottom-up approaches have been advocated in Africa to address the top-down organization of services and some of these delivery and governance challenges. This has been a major discourse in the zoonoses literature, albeit it is questionable how much it has moved beyond mere rhetoric. Bernard Bett, again from ILRI, echoed this widespread opinion:

… I think if we started to work within that One Health paradigm from bottom-up, involving people on the ground … then we can identify areas that require support and work on those … [These] will be taken up much faster than if we just identified things on our own and tried to take them back to decision makers. (See electronic supplementary material.).

There are examples to draw upon; for instance, decades of non-government organization (NGO)-supported initiatives to train community-based animal health workers (CAHWs), which were very successfully used in rinderpest elimination. Participatory livestock development schemes have also been widely supported and, to a much lesser degree, participatory disease surveillance networks [35,36]. Similar efforts have been done in the human health and conservation sectors, with community-based health worker networks [37] and community-based conservation campaigns. Clearly, one of the major lessons to have come from the West African Ebola epidemic is the importance of engaging and working with the existing mitigation practices of communities in disease control [38].

However, large-scale community-based interventions for the prevention and control of endemic zoonotic diseases, or ‘pre-epidemic’ emerging zoonoses, are few in Africa; the lack of implementation raises questions about feasibility and design. An increasing number of anthropological studies have revealed how complex social, cultural, political, economic and environmental determinants influence endemic zoonoses interventions. This includes studies on mass dog vaccination for rabies in Tanzania [39], community mobilization for locally appropriate sanitation infrastructure to prevent cysticercosis in Zambia [40], local use of veterinary drugs for trypanosomiasis and tick-borne diseases in Uganda [41] and the discarding of hydatid cysts in slaughterhouses in Morocco [42]. Other studies have explored risk behaviours for emerging zoonoses, such as bushmeat hunting and contact with primates in forest landscapes [43]. These studies all raise the important question: if One Health demands local engagement, how can policies and projects, adapted to local contexts, be effectively scaled-up and sustained? Answering this will require thinking not only about the context of implementation, but also about the prioritization process.

### (c) The politics of prioritization

Many of the issues with research and action discussed so far are embedded within the logic and structure of dominant policy and governance regimes. This underpins the observation that the simple provision of ‘evidence’, or more knowledge, has not led to large-scale change in driving One Health forward. As one of our interviewees (Delia Grace) stated:

What will make politicians change their minds? That is an interesting area … but … it may be that simple things like evidence or financial information is not very compelling. So what should we do? (See electronic supplementary material.)

In its socio-political context, One Health aspires to change not only ways of working but also existing governance and network relationships, and so influence power and politics [1]. Narratives about problems and solutions become important in steering political support, shaping discourse and framing the boundaries for actions, with significant implications. Through discourse analysis of policy documents and a wide range of key informant interviews at the global level, Galaz *et al*. [17] divided current One Health narratives into four divergent storylines: the ‘integration’ narrative; the risk and surveillance ‘outbreak’ narrative; the ‘economic benefits’ narrative; and the ‘local context’ narrative. They argue that, although these often overlap in practice, a dominant emphasis continues to be on biosecurity, epidemic crisis management, emergency funding and novel technologies [1,2]. The local context narrative that highlights local understandings, agency, capacities, priorities and conditions and attempts to empower alternative voices, does not, as the authors conclude, ‘synchronize with established models of resource mobilization and interests.’ [17, p. 34]. However, prioritizing a focus on local systems is imperative if a One Health approach to zoonotic disease is going to be more widely implemented.

This problem needs to be understood within the long-critiqued donor and NGO bureaucratic and administrative order, which support capacity development projects in Africa. These often involve short-term project cycles, strict pre-defined deliverables, outside experts and the continual jump towards the next big trend [44]. They have also tended to prioritize, as widely discussed in the humanitarian and development literature, the perspective and priorities of the Global North over those of the Global South [2]. Fragmented institutional landscapes, inhabited largely by NGOs, create holes in governance and accountability, and long-term support needed to effectively build systems falls to the wayside. Abramowitz [45] discussed this in relation to Liberia's post-conflict health system transition and reconstruction. She vividly documents how the NGO-ization of health systems created a set of conditions for the amplification of the Ebola virus disease outbreak in West Africa. These gaps in governance maintain systemic weaknesses and vulnerabilities that can act as ‘vectors’ for epidemic spread, but also for more hidden, endemic disease challenges.

Are current One Health activities in any way challenging these trends? The answer seems to be: very minimally, if at all. Based on a network analysis of knowledge flows around Nipah virus, Valeix *et al*. [46] showed that, despite the emphasis on collaboration and interdisciplinarity in research, scholars from the Global South remain marginalized as boundary partners and nodes at the global level, with limited capacity to shape the agenda. This speaks poorly for the wider socio-political dynamics discussed above.

Social science analysis of localized One Health activities is important to understand the constraints for action and how to move beyond them [1]. Ducrotoy *et al*. [47] provided a unique exploration under a flagship EU-funded neglected bacterial zoonoses (anthrax, brucellosis and bovine tuberculosis) project in Nigeria. They discussed how the underlying political and professional research interests of the academic partners, together with pre-determined project deliverables, constrained local action among marginalized Fulani pastoralists. Contrary to the dominant narrative that Fulani are ‘backward nomads’ that spread brucellosis through ‘poor’ husbandry practices, the researchers discovered that local livelihoods and migration patterns had the reverse effect: they helped mitigate disease spread [47]. Cultural and professional worlds were related over time through a process of ‘muddling through’ with the community. However, the project ended just as the researchers developed the local knowledge, networks and capacity needed for more proactive grassroots and higher-level policy engagement. Further grants were written to translate the research findings into local systems of action, but the funding was not forthcoming. The activities produced little immediate benefit for the Fulani.

In reality, moving policy and funding models away from the emergency mode, and into building resilient and integrated local systems that link research, action and policy will prove difficult, especially in an arena of competing health dollars and priorities. The Commission on a Global Health Risk Framework for the Future (CGHRF), convened by the US National Academy of Medicine after the West African Ebola epidemic, estimated that pandemics in the twenty-first century will cost the global economy some $6 trillion [48]. To avoid such catastrophe, they recommend spending $4.5 billion per year (or 65 cents per person worldwide), a ‘bargain’, largely on upgrading public health infrastructure and new pathways for drug, vaccine and product development [48]. Such initiatives emphasize the need to address the systemic weaknesses, and the insufficient financial and policy support, needed to strengthen countries' core capacities under the WHO's International Health Regulations (IHRs) [49]. While the IHRs are clearly important, Galaz *et al*. [17] and others have argued that by relegating the ‘local context’ narrative to an amorphous and often ill-defined field of ‘risk communication’ insufficient attention has been given to community-level social, behavioural and structural dynamics.

## 3. Toward solutions? Linking better research-policy-action in Africa

So, how can we move beyond mere rhetoric of change and link better zoonoses research, policy and action in Africa? How can we address the challenges in practical terms, in ways that benefit the health of local people, animals and the environment? Enacting positive change will be grounded in highly context-specific factors, problems, alliances, negotiations and entanglements. Some issues will certainly be easier to rally around and mobilize support for than others. Certain things may work in one context but not in others, and the more difficult issues may be the most pressing and important. Building on our analysis, here we discuss four strategies that could help unlock the potential for a One Health approach to zoonotic disease for the future.

### (a) Beyond the expert agenda

The first strategy is a more concerted effort for the co-production of knowledge. In their now classic book, *The New Production of Knowledge: The Dynamics of Science and Research in Contemporary Societies*, Gibbons *et al*. [24] distinguished between ‘Mode 1’ science, characterized by the hegemony of disciplines, the university and internal hierarchy of scientists, and ‘Mode 2’ science, which is applied, trans-disciplinary and accountable to societal challenges. This latter form of knowledge requires a re-orientation of the role of the scientists. With decades of experience in rabies and other endemic zoonotic diseases in Africa, Jakob Zinsstag, head of the Human and Animal Health Unit at the Swiss TPH, discussed this as follows:

People always say, ‘link science and policy’ but that's only a part of the piece. What we need is societal engagement … we must engage with all stakehold[ers] that are tied to a societal problem. This means communities, [government] authorities, technical experts, private industry. Anybody that is related to a problem should be engaged…as actors in the research. This is what we call trans-disciplinary research, which co-produces knowledge … There is an added-value to this knowledge generation that cannot come from the desk or lab of a scientist and that can only come from the field … The scientist becomes more a moderator of the process than just … a brain. (See electronic supplementary material.)

Hence, the repeated maxim that science needs to be linked to policy is overly simplistic as it assumes policymakers are passive recipients of knowledge, waiting for input from scientific experts [25]. Elizabeth Mumford, who works on building One Health capacity and policy for the Country Health Emergency Preparedness and IHR Department of the World Health Organization, further emphasized the need to link policymakers and their priorities into the research process:

It's not finding research and figuring out how to implement it. It's identifying gaps in knowledge, in issues, in … health systems … Then it's not a process of translating research into policy. The gap in policy exists and the research is created to fill that gap. (See electronic supplementary material.)

Co-creation of knowledge, however, will require challenging the expert agenda in order to highlight, engage and empower alternative voices and concerns. Vupenyu Dzingirai, from the Centre for Applied Social Sciences, University of Zimbabwe, discussed the importance of ‘putting people first’ in this process:

Too often researchers speak on behalf of people, [even] on the behalf of policymakers. And in so doing, [they] reify or distort the reality which they are trying to understand … To make research [more] effective [we should allow] people on the ground to speak for themselves … in ways that define clearly what their problems are and … what it is that they would need to do to resolve them … Let's let people speak for themselves and [One Health] will work. (See electronic supplementary material.)

Hence, more attention needs to be focused on the co-production and co-management, or democratization, of the research process for the true potential of One Health to be realized. This has been given some discursive significance in the One Health literature [1]. However, this needs to be accompanied by mechanisms and pathways for feasible socio-political action and change.

A number of interdisciplinary research consortiums have explored the process for broad-level stakeholder engagement for zoonoses in Africa in recent years, guided by a One Health approach; see Integrated Control of Neglected Zoonoses in Africa (http://www.iconzafrica.org) and Dynamic Drivers of Disease in Africa (http://steps-centre.org/project/drivers_of_disease/), for example. A major lesson emerging from these European–African consortia has been that getting different disciplines to work together, including social scientists, takes time, and that the types of broad action–research partnerships we have advocated for here are exceedingly difficult, but nonetheless possible. Efforts to improve pastoralist access to human and animal health services in Chad, through such broad-based One Health engagement over nearly two decades, is a good example of this potential [50].

Moving beyond the expert agenda is, of course, a fluid process that requires dedication, time and investment. As we discussed above, these more laborious processes can be risky for researchers dependent on grant funding cycles and donor demands; dependence on other stakeholders, who cannot be ‘controlled’, can generate unforeseen roadblocks for quick results. As researchers become more entangled in a network of local stakeholders, the politicalized nature of knowledge and its ability to challenge local systems of resource distribution—of land, livelihoods, health and administrative control—also becomes more pronounced. However, it is in these more long-term networks where the full value of democratized knowledge can help drive systemic change in ways that reconfigure human–animal–ecosystem relations, and build more resilient zoonoses preparedness, prevention and control.

### (b) National coordinating bodies and networks

A second strategy to moving One Health forward into the ‘real-world’ includes strengthening in-country and regional networks in ways that link academic and government partners and account for local political cultures. One Health advocates have promoted the concept through a variety of research partnerships, training programmes and changes in institutional and organizational networks. A major focus has been on capacity building, through offering new ‘One Health’ courses at universities, a variety of north–south exchanges and the establishment of research centres of excellence [51,52]. This includes regional networks, such as the Southern African Centre for Infectious Disease Surveillance (SACIDS), the One Health Central and Eastern Africa (OHCEA) consortium and the Pan-Africa One Health Platform on Neglected Zoonotic Diseases. However, the lack of inclusion of social science in these capacity building networks is problematic; it is also unclear how these research-focused efforts address the systemic challenges we have discussed in this paper.

Moving the agenda demands new forms of policy negotiation and involvement of different government ministries. Greater coordination and involvement of national government bodies and NGO networks would advance the One Health agenda. Addressing the reticence to build long-term capacity in African institutions would be an important step in the right direction [34]. The focus should not only be on research capacity training, but also on examining different models for policy change, and different mechanisms for coordination, while considering existing country and regional contexts. One example is Kenya's inter-sectoral Zoonotic Disease Unit (ZDU), which has developed and begun to implement a National Rabies Control Strategy, while also initiating various studies on other pathogens, responding to outbreaks and developing epidemic preparedness plans.

Novel pathways need to take into account how African political systems operate as a basis for driving change. This has often been explained, in the political science literature, with reference to ‘clientelism’ and ‘neo-patromonialism’. But such processes can be much more complex. Bierschenk and de Sardan [53], well-known anthropologists in French West Africa, have explored the routines of ‘work’ in the African ‘state apparatus’, and highlighted how competing actors, organizations and normative ideas shape decisions and policy. This is done through:

… routines, compromises, make-shift solutions and *bricolage* … the construction sites of overlapping projects led by different actors … [revealing] both the incompleteness of state-building processes and the heterogeneity and (always) improvised nature of statehood. [53, p. 6]

The notion that statehood and policy are *improvised* is relevant to efforts to advance One Health. It points to the fluid, often ad hoc, co-production of policy between different actors and interests. To advance One Health, we need to ‘go with the grain’, developing tactics that push reform through a process of, as Bierschenk & de Sardan [53] state, *bricolage.* Those with an interest in advancing a new agenda need to do so by generating political support, positioning themselves in ways that appeal directly to policymaker interests. Again, the co-production knowledge process is relevant here. As Steve Osofsky, senior policy advisor at the Wildlife Conservation Society, stated:

We don't design our science in a vacuum, we try and work with decision makers … to figure out what is the information gap that they're perceiving – what information do they need to influence policy – and to have them part of the science process. So it's a continuum from science, to policy, to action … Having involvement of the decision makers … from the very earliest stages makes all the difference because by the time you have recommendations, they've been a part of the process all along. (See electronic supplementary material.)

Discussions around research evidence has occupied a great deal of effort in One Health, but advancing One Health demands engaging in ‘political entrepreneurship’; facilitating mechanisms and spaces between researchers, governments and civil society to co-create knowledge and policy is imperative.

### (c) Building on ‘what works’ in the region

A third strategy involves following a ‘working-with-what-works’ approach. Delia Grace from ILRI succinctly defined this as follows:

There is always a tendency to want the new initiative or new idea … instead of wanting to … have all of our laboratories … [linking data] via satellite because that is really cool [for example] … why not take [the example of a One Health laboratory system successfully used in Canada] and [apply it] to the Caribbean, France, Kenya [and beyond]. (See electronic supplementary material.)

This quote recognizes that, in many instances, we know what ‘best practices’ benefit ecosystems, animals and people's health. What is missing is the political will to effectively translate them at scale, and with sufficient attention to local and national contexts. What is less often discussed is that many of these interventions are not biomedically orientated but involve a variety of social determinants that mediate risk factors and behaviours for animal and human health [1].

Often, social determinants are intimately interrelated, and building local systems of action requires interspecies improvements and interventions that link with the development concerns of the community and district government. The Ugandan NGO, Conservation Through Public Health (CTPH), serves as a good, practice-based example of this at a local level. Director of CTPH, Gladys Kalema-Zikusoka, discussed the approach as follows:

We set up Conservation Through Public Health … when we had [Scabies Skin disease in] … mountain gorillas traced to people living around [Bwindi Impenetrable National Park, Uganda] … And we realized that we could not conserve and protect the gorillas without thinking about the health of the people who they share a habitat with … (See electronic supplementary material.)

With a global network of supporters, CTPH has successfully integrated gorilla conservation with broad-based public health improvements in and around national parks in Uganda since 2005 [54]. CTPH's work in Bwindi has involved setting-up a Village Health and Conservation Team network, which has resulted in improved community hygiene and sanitation, better treatment of infectious disease and family planning methods, reduced poaching and greater protection of gorillas [54]. Investing in scaling-up this approach to other contexts would be a worthwhile investment for conservation and development.

To do so, better documentation and learning of the contextual details involved in ‘success stories’, which is currently very sparse in the literature, are needed. Baum [55], for example, has highlighted how the lack of quantitative evaluation of One Health programmes handicaps efforts to scale-up proven strategies. In Europe, the Network for Evaluation of One Health (http://neoh.onehealthglobal.net) is a broad-based network aimed at plugging this gap and linking knowledge to policy and action. This may serve as a good model for future African-based efforts.

### (d) Storylines: reframing the problem to influence decision-makers

Lastly, a careful thought needs to be given to the ways in which the problem of zoonoses are framed, and how narratives about the problem and solutions can be articulated in ways that build support, interest and investment to address the systemic weaknesses discussed above. Packaging a ‘storyline’ narrative demands, in some sense, asking the important question: what gets people moving? Clearly, the major impetuous driving zoonoses investments and control are the linkage to human health benefit. Peter Daszak, President of the EcoHealth Alliance, reflected on this opinion:

It is a tough job to try and translate what is happening in the science to policymakers and the public … I think we need to talk in really simple terms where there is real benefit for people. People think about their own health more than really they think about wildlife or conservation … We need to frame our message more simply and more directly to public health. (See electronic supplementary material.)

Appealing directly to public health is one major reason for the dominant attention to pandemic threats. History (Black Plague, Spanish influenza, HIV/AIDS, etc.) has shown that this is clearly important. Such framing captures the interests of politicians and global institutions, and can lend itself to be sold on the market of international funding. But while the priorities of the global health security agenda are certainly important, there are other pathways and storylines that should also be engaged, and that include a central appeal to public health benefit. Steve Osofsky discussed the need for a more holistic framing of ecosystems and health, for example:

[One Health has] always been much broader than infectious disease … It is about our global systems and the fact that we are altering virtually all the world's biogeochemical cycles … and all of those activities have real consequences … for health. If One Health … is going to succeed, it's largely got to capture the public health impacts of … [these many] ecological changes … [these impacts] can't remain in the realm of externality. That's been our primary problem. There are actors who are causing these degradative changes; they are not bearing the costs. The global commons is bearing the costs. And until we have an economic system that captures those consequences, those public health losses … we will not be able to really influence policy quickly enough. (See electronic supplementary material.)

In this sense, ecosystem degradation, for example gorilla extinctions (to go back to the example of CTPH in Uganda), have an intrinsic value that needs to be accounted for. Our understanding of what constitutes ‘human health’ needs to be widened to include the deep interrelatedness of human well-being, not only ‘disease’, to animals and the environment.

In the case of zoonoses, it is clear that risk factors for exposure are influenced by a vast array of ecosystem factors, animal and human behaviours and political economy dynamics [1,22]. Drawing attention to these processes is important, as are efforts to include them in the planning and policy cycle through, for example, scenario planning [23]. Another area where further work is needed is on the relationships between endemic zoonoses and food (in)security and human nutrition [10]. Lastly, some scholars have argued that, if One Health is to truly succeed, the narrative needs to be broadened in order to question larger systems of power and influence that underpin the root social and ecological causes of vulnerability to emerging pandemics [1,13,56]. Importantly, this includes addressing the deficiencies in neoliberal ideology and the market-based economy in structuring the ecological crises and lack of governance of the ‘global commons’ that underlay disease emergence and transmission [14].

The importance of alternative framings of complex societal and environmental problems has been widely discussed in the climate change and environmental movements, and lessons could be learnt from them. There has been some progress in this regard, as seen in a proliferation of expert commissions and consultations on the relationships between human health and ecosystems, from reports on biodiversity and human health [57]. Zoonoses feature prominently in these debates.

However, such assessments may represent ‘talking-shops’ if they are not readily accompanied by concerted efforts to address the politics of funding [1] and the participation of diverse stakeholders and interests in the production of knowledge, action and policy. The inherent uncertainty of where and when diseases will emerge drives what Waldman *et al*. [58], exploring narratives of bat-associated zoonoses in Ghana, have called a ‘politics of precaution.’ Evidence gaps are highlighted to justify inaction, and different sectoral perspectives and framings block action. Pandemic prevention, in this sense, while intellectually appealing, appears to be wholly unrealistic unless it becomes framed more as an integral part of wider poverty alleviation efforts and system strengthening of veterinary, public health and conservation services. One concrete and important step forward has included efforts to establish mechanisms, similar to environmental risk assessments, to influence private companies in the oil, mining, large-scale agriculture and forestry sectors [59] to reduce the impact of their extractive activities on health and ecosystems. But much more is clearly needed.

## 4. Conclusion

In this paper, we have argued that the current challenges to realizing One Health in the ‘real-world’ for the control of zoonotic diseases in Africa, both endemic and emerging, are related to a number of systemic challenges in research, action and policy. Moving beyond the current rhetoric of One Health will demand engaging in multiple tensions and divergences in power, knowledge construction, material resources, norms and values that mediate political action and social change. Ultimately, this suggests that One Health has an important socio-political aspect, one that aims to challenge accepted orthodoxies. Advancing One Health, therefore, will not only require more collaboration and integration among scientific experts but, as we have argued, a fundamental re-orientation: a democratization of science and public policy.

## Supplementary Material

Locations of video interviews

## End notes

1 It is important to note that the term ‘One Health’ is defined differently by different people [17]. For some, it is synonymous with zoonotic disease research and control, where the animal and human health sectors collaborate to address specific diseases at the interface among humans, animals and the environments they live in. But for many others, the term has a much broader reference and applicability, cutting across a more diverse number of issues—from antimicrobial resistance to marine ecosystems. In this paper, when we use the term ‘One Health’, we are referring to the former, zoonoses-specific definition.

2 Our selection of participants was based on the panel discussions at the symposium, which was largely ‘researcher-based’, including a panel on the theme of engaging research with policy and action. The video interviews were meant to be short (approximately 5 min) and succinct reflections, and not in-depth interviews. All participants were provided with the two questions in advance. All of the 11 interviewees were then invited to comment on the original analysis. Most did, and became co-authors on this paper, providing additional comments and reflections on the major themes.
