# Supplementary material for: Engaging research with policy and action: what are the challenges of responding to zoonotic disease in Africa?
Source: Philos Trans R Soc Lond B Biol Sci. 2017 Jun 5;372(1725):20160172. doi: 10.1098/rstb.2016.0172 (PMC5468697; doi:10.1098/rstb.2016.0172)
Supplement: Locations of video interviews [file rstb20160172supp1.docx]

**Video Interviews – Supplementary Files**

1. Interview with Peter Daszak

<https://www.youtube.com/watch?v=2fqTr6mpjqE>

2. Interview with Bernard Bett

<https://www.youtube.com/watch?v=EBw5Ln4Aamc&feature=youtu.be>

3. Interview with David-Waltner-Toews

<https://www.youtube.com/watch?v=ZBEOE4i4grY&feature=youtu.be>

4. Interview with Delia Grace

<https://www.youtube.com/watch?v=rBq9h3pL0jw&feature=youtu.be>

5. Interview with Elizabeth Mumford

<https://www.youtube.com/watch?v=4t9WIQqrlKU&feature=youtu.be>

6. Interview with Gladys Kalema-Zikusoka

<https://www.youtube.com/watch?v=ae4PPbOJ7AU&feature=youtu.be>

7. Interview with Jakob Zinsstag

<https://www.youtube.com/watch?v=dAMYXjiKyo0&feature=youtu.be>

8. Interview with Kate Jones

<https://www.youtube.com/watch?v=vsJG3btREzE&feature=youtu.be>

9. Interview with Katinka de Balogh

<https://www.youtube.com/watch?v=p5O_GiOAdds&feature=youtu.be>

10. Interview with Steve Osofsky

<https://www.youtube.com/watch?v=K7c8tgJ8Aqg&feature=youtu.be>

11. Interview with Vupenyu Dzingirai

<https://www.youtube.com/watch?v=Oo-7Miwrro0&feature=youtu.be>
